# Neural basis of uncertain cue processing in trait anxiety

**DOI:** 10.1038/srep21298

**Published:** 2016-02-19

**Authors:** Meng Zhang, Chao Ma, Yanyan Luo, Ji Li, Qingwei Li, Yijun Liu, Cody Ding, Jiang Qiu

**Affiliations:** 1 Key Laboratory of Cognition and Personality (SWU), Ministry of Education, Chongqing, China; 2 Faculty of Psychology, Southwest University, Chongqing 400715, China; 3 Department of Psychology, Xinxiang Medical University, Henan 453003, China; 4 School of Nursing, Xinxiang Medical University, Henan 453003, China; 5 Shanghai Mental Health Center, Shanghai 200030, China; 6 Department of Psychiatry, University of Florida, 100 Newell Drive, Gainesville, FL 32610-0256, USA

## Abstract

Individuals with high trait anxiety form a non-clinical group with a predisposition for an anxiety-related bias in emotional and cognitive processing that is considered by some to be a prerequisite for psychiatric disorders. Anxious individuals tend to experience more worry under uncertainty, and processing uncertain information is an important, but often overlooked factor in anxiety. So, we decided to explore the brain correlates of processing uncertain information in individuals with high trait anxiety using the learn-test paradigm. Behaviorally, the percentages on memory test and the likelihood ratios of identifying novel stimuli under uncertainty were similar to the certain fear condition, but different from the certain neutral condition. The brain results showed that the visual cortex, bilateral fusiform gyrus, and right parahippocampal gyrus were active during the processing of uncertain cues. Moreover, we found that trait anxiety was positively correlated with the BOLD signal of the right parahippocampal gyrus during the processing of uncertain cues. No significant results were found in the amygdala during uncertain cue processing. These results suggest that memory retrieval is associated with uncertain cue processing, which is underpinned by over-activation of the right parahippocampal gyrus, in individuals with high trait anxiety.

Anxiety may be triggered by stressors, which may produce persistent fears and create a stream of negative thoughts that can gradually make people more anxious. Anxiety disorders are characterized by a state of apprehensive expectation, hyperarousal, vigilance to threat cues, fear, and avoidance behaviors^1^. Anxiety includes fear of uncertainty, and some studies have reported that the uncertainty, especially in relation to potentially negative stimuli, often provokes anxiety. Furthermore, it has been hypothesized that anxious individuals have particular difficulty tolerating uncertainty^2^,^3^,^4^. A study by Williams *et al.* makes the neural connection between anxiety and uncertainty by showing that increased amygdala activity may play a crucial role in processing uncertain information in preadolescent children with anxiety disorders^5^. To date, it is unclear which neural circuits process uncertain cues in individuals with non-clinical anxiety. Understanding the neural basis of uncertainty in non-clinically anxious individuals will provide a broad picture of the neuroscientific basis of anxiety disorders.

Trait anxiety is a personality trait that reflects an individual’s disposition for an anxiety-related cognitive and affective processing bias^6^. Trait anxiety, as a stable predisposition in normal individuals, is often considered to be a risk factor for anxiety disorders and other psychiatric illnesses^7^,^8^,^9^,^10^. Moreover, we recently investigated the correlations between trait anxiety scores and regional gray matter volumes (rGMV) and regional BOLD baseline – with the amplitude of low frequency fluctuations (ALFF) as the index – in 383 university students. We found that anxiety (a) was negatively correlated with rGMV in the right middle occipital gyrus, (b) was positively correlated with the ALFF in the right supplementary motor area and the bilateral superior frontal gyrus, and (c) was negatively correlated with the ALFF in the thalamus and left cerebellum. This experiment, which was conducted with a normal sample, found that individuals with high trait anxiety showed attenuated image processing on a consciousness level (cognitive processing bias) and exhibited stronger induced sensibility and over-processing ability of the relationships (emotional processing bias) (submitted). Given that the results of uncertain stimuli (or signals) can be interpreted as maintaining avoidance of a potential threat, vulnerability to anxiety during uncertainty may reflect greater memory retrieval in anxious individuals. Such findings suggest that the processing of uncertain cues in individuals with high trait anxiety is disordered.

Therefore, in order to explore the neural basis of uncertain cue processing in individuals with high trait anxiety, we conducted an fMRI experiment to characterize the uncertainty of fear stimuli. The neutral and fear stimuli were all pictures of objects. In daily life, an individual’s memory and expectations will influence their experience. So, to test the neural circuits of uncertainty, we employed a learn-test paradigm (see Figs 1 and 2). This experimental paradigm can help us explore the brain activity pattern during uncertain cue processing and the subsequent experience of future events.

## Methods

### Participants

Thirty-five individuals (23 females; mean age = 21.46 years old) participated in the study. The participants were undergraduate or postgraduate university students in Southwest University, China. They were recruited either through advertisements on a bulletin board in Southwest University or introduced by persons who participated in previous experiments in our laboratory. All participants were screened using the Structured Clinical Interview of the DSM-IV, which was performed by two well-trained and experienced Ph.D. candidates in the Faculty of Psychology of Southwest University. Thus, participants who met one of the following lists would be out of this experiment: substance abuse disorders, neurologic disease, psychiatric disorder, histories of neurological or psychiatric illnesses, visual difficulties, had conditions which made them unsuitable for scanning, such as head trauma, taking medications that may change brain function, a history of loss of consciousness, pregnancy, or breast-feeding. All participants gave their written informed consent in accordance with the Declaration of Helsinki^11^. The institutional ethics committee of the Southwest University Brain Imaging Center Institutional Review Board approved the study protocol. The experimental methods were carried out in “accordance” with the approved guidelines.

### Behavioral assessments

Before the formal experiment, another twenty participants rated all the neutral and fear stimuli for intensity on a scale from 1 to 9 (1 being the least intense emotion, and 9 being the most intense emotion) for each of the basic emotions (happiness, surprise, sadness, fear, anger, disgust)^12^, and for emotional valence, arousal, and dominance on scales from 1 to 9.

Each person who participated in the formal experiment was evaluated for trait anxiety using the Trait Anxiety Inventory (T-AI). The T-AI is a self-report questionnaire that consists of 20 items that measure anxiety-related trait personality^6^,^13^. The T-AI is valued for its high internal consistency and its test-reliability, which ranges from 0.73 to 0.86 across multiple samples^6^.

### Learning task and test

**Figure 1:**
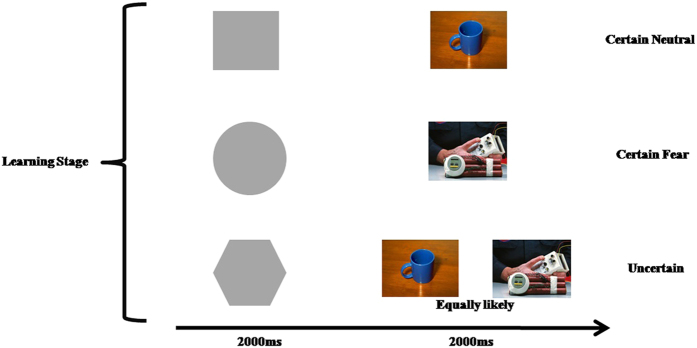
The learn stage of the experiment paradigm.

**Figure 2:**
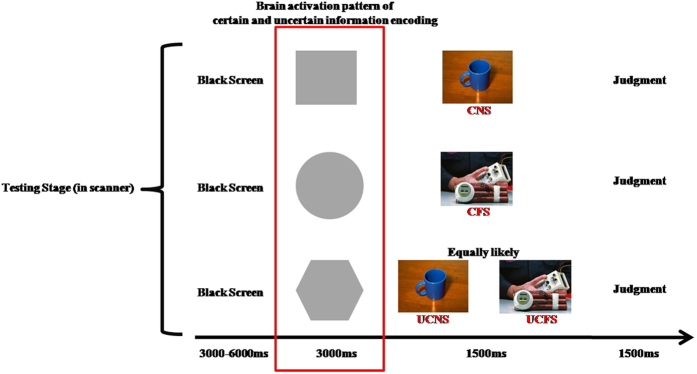
The test stage of the experimental paradigm.

As shown in Figs 1 and 2, the task consisted of two phases. The first phase was a learning stage that was conducted before scanning. In this phase, the participants were asked to learn the relationship between the neutral shape cues and pictures of objects presented on a computer screen. This phase consisted of 60 trials, with 20 trials in each of three conditions (a certain neutral condition, a certain fear condition, and an uncertain condition), with each trial consisting of an abstract stimulus (2000 ms) and a picture of objects (2000 ms). After learning stage, the participants were asked to perform a test which a neutral cue appeared on the screen, the participants needed to predict the subsequent stimuli’ emotional valence. The participants could not perform the second phase, until they correctly learned the relationships between the neutral shape cues and the objects.

In the second phase, which was conducted in the MRI scanner, the participants were asked to decide whether the object that was presented after the neutral shape cues was an object they observed during the first phase. This phase was consisted of 72 trials, with 24 trails in each condition: the certain neutral condition (CNC), the certain fear condition (CFC), and the uncertain condition (UNC). Twelve objects were chosen from each condition in the first phase for use in the second phase. The time course of a single trial is illustrated in Fig. 2.

### MRI data acquisition

A 3.0-T Siemens Trio MRI scanner (Siemens Medical, Erlangen, Germany) and an eight-channel phased array coil were used to acquire high-resolution T1-weighted structural images (repetition time = 1900 ms; echo time = 2.52 ms; inversion time = 900 ms; flip angle = 9 degrees; resolution matrix = 256 × 256; slices = 176; thickness = 1.0 mm; voxel size = 1 × 1 × 1 mm^3^). T2*-weighted echo planar images also were obtained (25 slices, 3 mm × 3 mm × 4 mm voxels, TR = 1500 ms, TE = 30 ms, flip angel = 75°, FOV = 192 mm × 192 mm).

## Data analysis

### Behavioral data analysis

First, the percentages of correct answers on the memory test were subjected to one-way analysis of variance (ANOVA). We applied the theory of signal detection to the memory test.

According to the signal detection theory, a picture of object which was chosen from the first phase appears, and if the participants make a correct judgment, then marked as HIT, if the participants make a wrong judgment, then marked as MISS; a picture of object which was not chosen from the first phase appears, and if the participants make a correct judgment, then marked as CORRET REJECTION, if the participants make a wrong judgment, then marked as FALSE ALARM; then The P(H) and the P(FA) in the CNC, CFC, and the UNC were analyzed by the following formula:


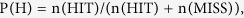



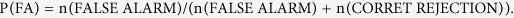


The P(H) and the P(FA) in the CNC, CFC, and the UNC were translated to O(H) and O(FA) using PZO translation. Then, the likelihood ratio (β) in the CNC, CFC. and the UNC were analyzed by the following formula:


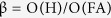


higher β values (the likelihood ratio or decision criteria, the more the β is, the more strict the criteria is) indicates worse memory performance in this study. The βvalues in the three conditions were subjected to one-way ANOVA. All *p*-values were corrected using the Bonferroni adjustment. Finally, correlations were performed on the trait anxiety scores and the percentages of the β values in the three conditions.

### fMRI data analysis

The focus of the analysis was the BOLD level of the different neutral shape cues. The data analysis was performed using SPM8 software from the Wellcome Department of Cognitive Neurology, London (SPM8, www.fil.ion.ucl.ac.uk/spm/), which was implemented on MatLab 7.10.0 R2010a (MathWorks, Natick, MA). All the analyses were started from the appearance of the abstract signal. Scans were slice-time corrected to the thirteenth slice, then realigned and normalized into standard Montreal Neurological Institute (MNI) space via 12-parameter affine transformation. Finally, all data were smoothed with a 6 mm full width at half maximum (FWHM) Gaussian kernel, and filtered (high-pass filter set at 128 s, low-pass filter achieved by convolution with the hemodynamic response function). After preprocessing, the statistical analyses for each individual participant were based on a fixed-effects general linear model (GLM) and analyses on the group level were based on a random-effects model. The resulting images had cubic voxels of 3 × 3 × 3 mm. The BOLD responses were modeled as events convolved with the canonical hemodynamic response function in SPM8. For each condition (CNC, CFC, and UNC), all trials were averaged to estimate BOLD responses.

In the group random-effects (second-level) analyses, participant-specific linear contrasts of the parameter estimates were entered in a series of one-sample *t*-tests, each constituting a group-level statistical map. Our main contrasts of interest were BOLD signals in response to different neutral shape cues to assess the main effect of conditions between UNC and CNC, and between UNC and CFC (FDR corrected, *p* < 0.05, voxel size 50). This contrast was used to identify the uncertain cue processing regions of the brain.

Moreover, we used REST software (http://www.restfmri.net/forum/REST V1.7) to extract the brain region signal of uncertainty (the neutral shape cues in the uncertain condition). Then a correlation between the trait anxiety scores and the brain region signals was conducted.

## Results

### Behavioral results

The fear stimuli were rated as evoking significantly more negativity, more arousal, more dominance, and more fear than the neutral stimuli (see Table 1).

One-way ANOVAs of the percentages of correct answers showed a significant main effect of conditions, *F*(2, 102) = 12.102, *p* < 0.001. Multiple comparisons showed that the percentage of correct answers was lowest in the CNC (*p* < 0.001), and that there was no significant difference between the percentage in the CFC and the UNC. Furthermore, ANOVAs of the β values showed a significant main effect of the three conditions, F(2, 102) = 9.775, *p* < 0.001. Multiple comparisons showed that the β values in the CNC was the largest of the three conditions (*p* < 0.01), and there was no significant difference between the β in the CFC and the UNC (see Table 2). There was no significant correlation between the trait anxiety scores and the percentages or the β values in CNC, CFC, and the UNC.

### Functional brain activity of UNC contrast CNC

**Figure 3:**
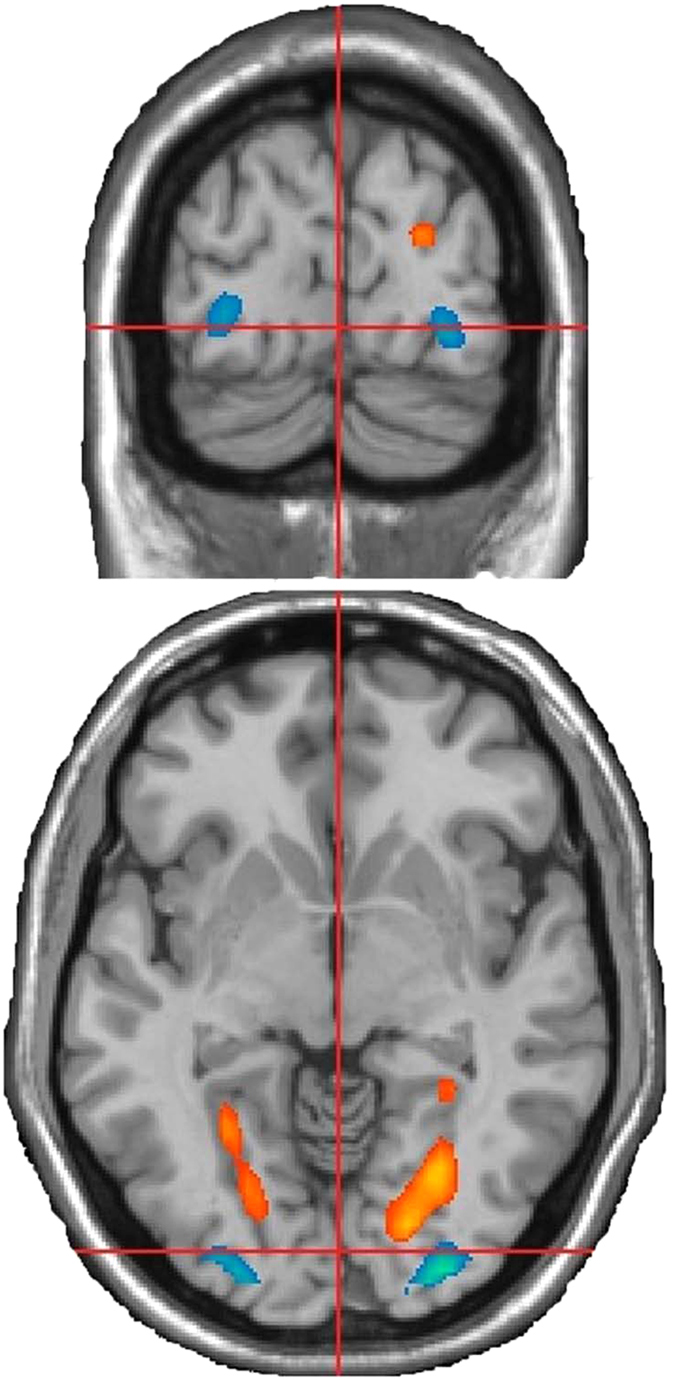
Brain areas of significant brain activation differences between UNC and CNC (p < 0.05, corrected with FDR).

Contrasts were made between the UNC and CNC using random-effects models. First, the contrast of the UNC versus the CNC showed activity in the following areas (FDR corrected, *p* < 0.05, voxel size 50): the left lingual gyrus, bilateral fusiform gyrus, and right parahippocampal gyrus. Second, the contrast of the CNC versus the UNC showed activity in the bilateral inferior occipital cortex (FDR corrected, *p* < 0.05, voxel size 50)(See Table 1 and Fig. 3).

### Functional brain activity of UNC contrast CFC

**Figure 4:**
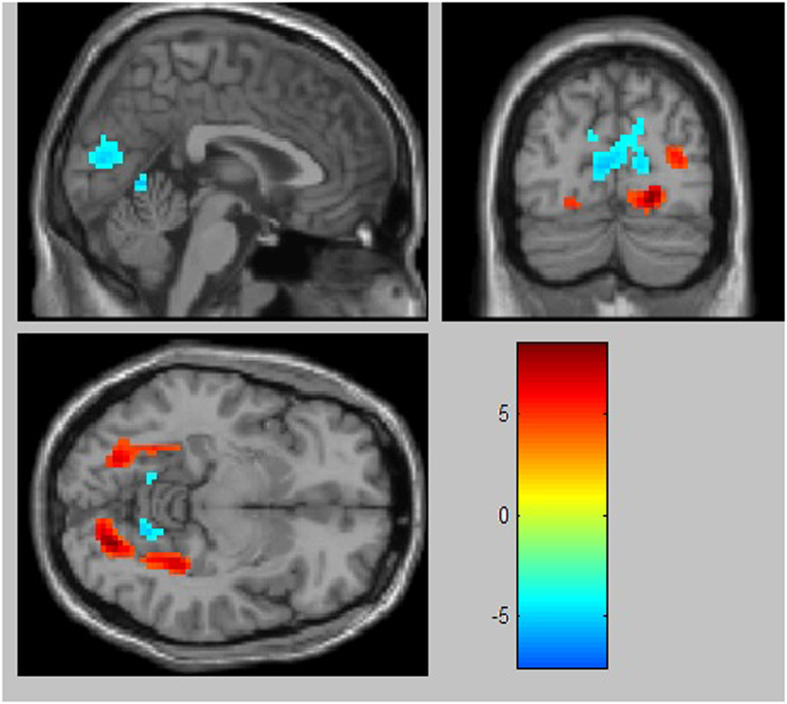
Brain areas of significant brain activation differences between UNC and CFC (p < 0.05, corrected with FDR).

Contrasts were made between the UNC and CFC using random-effects models. First, the contrast of the UNC versus the CFC showed activity in the following areas (voxel-wise threshold of *p* < 0.05, FDR corrected, cluster size 50): the bilateral middle occipital cortex, left superior occipital cortex, right lingual gyrus, left fusiform gyrus, and the right parahippocampal gyrus. Second, the contrast of the CFC versus the UNC showed activity in the following areas (voxel-wise threshold of *p* < 0.05, FDR corrected, cluster size 50): the right lingual gyrus, left calcarine gyrus, and the left cuneus (See Table 3 and Fig. 4).

### Correlations between brain regions of uncertainty and trait anxiety

**Figure 5:**
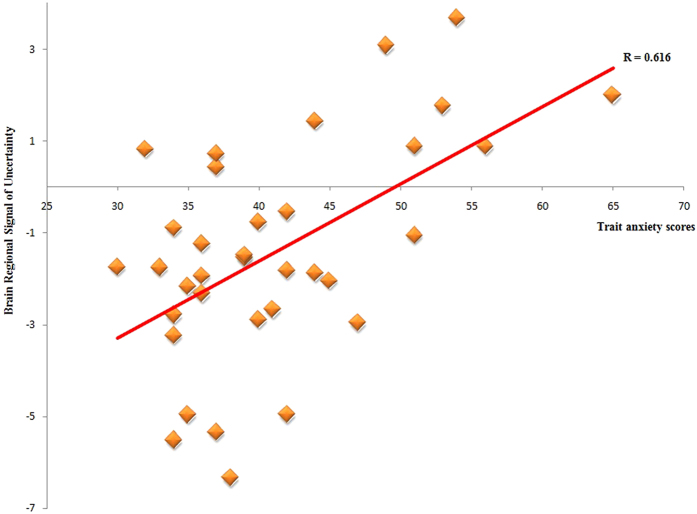
Correlation between the right parahippocampal gyrus signal of uncertainty condition and the trait anxiety scores.

After correcting for sex and age, trait anxiety scores had a significant positive correlation with the brain region signal of uncertainty in the right parahippocampal gyrus (*r* = 0. 62, *p* < 0.001) (See Fig. 5). The p-values were corrected using the Bonferroni adjustment (p < 0.05*1/n).

## Discussion

In the present study, we explored brain activity patterns during uncertain encoding and their relation to individual differences in trait anxiety. The behavioral results showed that the UNC was similar to the CFC, which suggests that there was a cognitive bias under uncertainty. The fMRI results showed that the visual cortex, bilateral fusiform gyrus, and the right parahippocampal gyrus were active during the processing of uncertain cues. Moreover, we found that trait anxiety was positively correlated with the BOLD signal of the right parahippocampal gyrus while the uncertain cue was being encoded. We discuss the implications of these results in the following section.

First, the visual cortex, including the bilateral lingual gyrus, bilateral middle occipital gyrus and the left superior occipital gyrus, were active in the contrast of the UNC versus the CNC and the contrast of the UNC versus the CFC. Furthermore, activity was found in the left calcarine, the left cuneus, and the right lingual gyrus in the contrast of the CFC versus the UNC. Previous studies indicated that the visual cortex predicts conscious processing^14^. Carlson *et al.* reported that the lingual gyrus, which modulates spatial attention and fear processing, is connected with the fear network, and Lai *et al.* suggested that the lingual gyrus might be a part of the fear network^15^,^16^. Moreover, increased activity of portions of the visual cortex has been found to be involved in the neural processing of fear of rejection, social sensitivity, the social cognition^17^. Killgore and Yurgelun-Todd reported that activity in portions of the visual cortex is related to stress from social interactions, and some areas may regulate anxiety, vigilance, and cardiovascular system functions^18^,^19^. So, based on previous studies, the visual cortex, including the bilateral lingual gyrus, bilateral middle occipital gyrus, and the left superior occipital gyrus may play an important role in the sending sensory information through the sensory afferents to the parahippocampal gyrus.

Second, we found more activity in the bilateral fusiform gyrus in the contrast of the UNC with the CNC and CFC. The fusiform gyrus is a brain region that is not only related to perceptual processing (face and other objects)^20^, but also related to emotional processing. Fairhall and Ishai conducted an fMRI study that found greater coupling between the fusiform gyrus and the amygdala when pictures of faces expressed emotions^21^. Moreover, Campbell *et al.* suggested that amygdala activity might occur together with an non-enhanced fusiform gyrus in both individuals with anxiety disorders and anxiety-prone individuals^22^,^23^. Straube *et al.* found the activity of the fusiform gyrus of patients with anxiety disorders was greater, relative to control subjects, during the processing of angry faces^24^. In the present study, the greater activity of the bilateral fusiform gyrus in the contrast of the UNC with the CNC and CFC might reflect the process of connecting the perception to the memory.

Third, greater activity of the right parahippocampal gyrus was found in the contrast of the UNC with the CNC and CFC, and a significant positive correlation was found between trait anxiety and the right parahippocampal gyrus signal in the UNC. Previous studies reported that activation of the parahippocampal gyrus might be related to the recovery of sensory experiences, as true memories have more details than false memories^25^,^26^,^27^. Moreover, false memories have never been found to produce activity in the parahippocampal gyrus when contrasted with true memories^28^,^29^. Some researchers also have suggested that true memories contain more sensory details, which is consistent with the fact that the parahippocampal gyrus mediates sensory processing^30^,^31^,^32^. In sum, Slotnick & Schacter suggested that parahippocampal gyrus activity is related to recollection to a greater degree than to familiarity^33^. Furthermore, the parahippocampal gyrus has a connection with the amygdala, which is a key brain region of emotion. Previous studies suggested that arousing stimuli increase the functional brain connection between the parahippocampal gyrus and the amygdala, and the amygdala plays a role in modulating emotional memory processing^34^,^35^. In the present study, the greater activity of the right parahippocampal gyrus in the contrast of the UNC with the CNC or CFC might involve the retrieval of more memories under unknown fear. In addition, the positive correlation between trait anxiety and the right parahippocampal gyrus signal suggests that highly anxious individuals may retrieve more memories automatically when they are in an uncertain situation.

Previous studies reported that individuals with high trait anxiety may present anxiety – related bias in emotional and cognitive processing that are considered risk factors for anxiety disorders and psychiatric disorders^7^,^8^,^9^,^10^. Our results in this study, consistent with the previous, indicated that individuals with high trait anxiety showed stronger sensibility, over-processing of relationships, and attenuated image processing on a consciousness level, and exhibited more automatic memory retrieve when they faced uncertain cues^36^. These features, which were different from those of normal subjects, may lead to excessive negative associations (anxiety disorders) and indulging in the past (depression and bipolar disorders). Furthermore, the results also indicated that it is important to develop a memory retrieval-extinction procedure for the individuals with high trait anxiety, or anxiety disorders, or other mood disorders, after they had experienced trauma. Moreover, as is generally known, unlike in depression and bipolar disorders, the principle feature of anxiety bias is fear of the future, especially of uncertainty. Excessive emotional memory retrieval caused by cues could lead to over worry in anxiety disorders, with indulging in excessive emotional memory retrieval caused by cues. Therefore, this paradigm may also be used to distinguish and predict individuals with high trait anxiety in the future.

Last but not least, in this study, we did not find activation of the amygdala in any contrast. The amygdale is an important brain region not only in anxiety disorders, but also in trait anxiety. There are two possible reasons which may explain the absence of amygdala activation. One reason for this may be the stimuli. Faces, especially fearful faces, are ideal stimuli because fearful faces reliably elicit an amygdala response^37^,^38^. Another reason is that the cue (neutral shape cues) involves recognition memory, and some memory experiments have shown that recognition of emotional memory often leads to activity in the parahippocampal gyrus, but notably, not the amygdala^39^,^40^. Furthermore, according to previous studies, we predict that the amygdale, or the insula, or the anterior cingulum cortex, which are involved in the negative stimuli, may over-activate during the image viewing in the non-clinical individuals with high trait anxiety when they face the uncertain negative pictures of objects^41^,^42^,^43^,^44^. There may be activation of these brain regions in uncertain shape cue processing. However, this idea will need to be tested.

## Conclusion

Individuals with high trait anxiety form a non-clinical group with a predisposition for an emotional and cognitive processing bias that is considered to be a pre-existing condition for psychiatric disorders. Anxious individuals tend to experience more worry under uncertainty. However, it is unclear what the neural circuits are that process uncertain cue in non-clinically anxious individuals. So, we explored brain activity patterns under uncertainty in trait anxiety. The results showed that the visual cortex, the bilateral fusiform gyrus, and the right parahippocampal gyrus were active during the processing of uncertain cues. Moreover, we found that trait anxiety was positively correlated with the BOLD signal of the right parahippocampal gyrus during uncertain cue processing. These results reveal the importance of memory retrieval for individuals with high trait anxiety in an unknown fear situation.

## Additional Information

**How to cite this article**: Zhang, M. *et al.* Neural basis of uncertain cue processing in trait anxiety. *Sci. Rep.* **6**, 21298; doi: 10.1038/srep21298 (2016).

## Figures and Tables

**Table 1:** The neutral and fear stimuli rating.

	VAL	Aro	Dom	Happy	Surprise	Sad	Fear	Anger	Disgust
Neutral	5.4 ± 0.7	3.2 ± 1.0	5.8 ± 0.8	2.6 ± 0.8	2.1 ± 0.9	1.8 ± 0.6	1.9 ± 0.4	1.7 ± 0.6	1.2 ± 0.2
Fear	3.1 ± 0.8	6.4 ± 1.0	6.1 ± 0.6	1.8 ± 0.5	3.2 ± 1.2	2.5 ± 0.9	4.8 ± 0.9	2.8 ± 1.1	1.9 ± 0.9

Note, VAL: emotional valence; Aro: Arousal; Dom: Dominance.

**Table 2:** The percentages and the β value in CNC, CFC, and UNC.

	Percentage (mean ± s.d.)	β value (mean ± s.d.)
CNC	0.61 ± 0.077	0.89 ± 0.046
CFC	0.70 ± 0.078	0.84 ± 0.045
UNC	0.69 ± 0.089	0.85 ± 0.063

**Table 3:** Brain areas of significant differences between random two of the three experiment conditions (UNC, CNC and CFC).

Brain regions	t-score	Talairach coordinates		
		x	y	z
UNC > CNC				
L				
lingual gyrus	6.87	−18	−81	−9
fusiform gyrus	6.90	−27	−66	−9
R				
fusiform gyrus	6.15	30	−57	−6
Rparahippocampal gyrus	4.47	33	−39	−9
CNC > UNC				
L				
inferior occipital cortex	9.17	−27	−93	−9
R				
inferior occipital cortex	8.12	27	−96	−6
UNC > CFC				
L				
middle occipital cortex	5.94	−27	−87	15
fusiform gyrus	6.47	−24	−72	−9
superior occipital cortex	4.99	−12	−99	12
R				
lingual gyrus	8.47	21	−75	−9
middle occipital cortex	6.92	30	−90	15
parahippocampal gyrus	7.77	33	−39	−12
CFC > UNC				
L				
calcarine	5.54	−6	−78	9
cuneus	5.47	−12	−84	30
R				
lingual gyrus	7.44	9	−60	0

Note: the threshold was set at p < 0.05 (corrected with FDR); L, Left; R, Right. UNC, uncertain condition; CNC, certain neutral condition; CFC, certain fear condition.
